# Autophagy Function and Benefits of Autophagy Induction in Models of Spinocerebellar Ataxia Type 3

**DOI:** 10.3390/cells12060893

**Published:** 2023-03-14

**Authors:** Maxinne Watchon, Luan Luu, Stuart K. Plenderleith, Kristy C. Yuan, Angela S. Laird

**Affiliations:** Center for MND Research, Macquarie Medical School, Macquarie University, Sydney, NSW 2109, Australia

**Keywords:** autophagy, polyglutamine, zebrafish

## Abstract

Background: Spinocerebellar ataxia 3 (SCA3, also known as Machado Joseph disease) is a fatal neurodegenerative disease caused by the expansion of the trinucleotide repeat region within the *ATXN3/MJD* gene. The presence of this genetic expansion results in an ataxin-3 protein containing a polyglutamine repeat region, which renders the ataxin-3 protein aggregation prone. Formation of ataxin-3 protein aggregates is linked with neuronal loss and, therefore, the development of motor deficits. Methods: Here, we investigated whether the autophagy protein quality control pathway, which is important in the process of protein aggregate removal, is impaired in a cell culture and zebrafish model of SCA3. Results: We found that SH-SY5Y cells expressing human ataxin-3 containing polyglutamine expansion exhibited aberrant levels of autophagy substrates, including increased p62 and decreased LC3II (following bafilomycin treatment), compared to the controls. Similarly, transgenic SCA3 zebrafish showed signs of autophagy impairment at early disease stages (larval), as well as p62 accumulation at advanced age stages (18 months old). We then examined whether treating with compounds known to induce autophagy activity, would aid removal of human ataxin-3 84Q and improve the swimming of the SCA3 zebrafish larvae. We found that treatment with loperamide, trehalose, rapamycin, and MG132 each improved the swimming of the SCA3 zebrafish compared to the vehicle-treated controls. Conclusion: We propose that signs of autophagy impairment occur in the SH-SY5Y model of SCA3 and SCA3 zebrafish at larval and advanced age stages. Treatment of the larval SCA3 zebrafish with various compounds with autophagy induction capacity was able to produce the improved swimming of the zebrafish, suggesting the potential benefit of autophagy-inducing compounds for the treatment of SCA3.

## 1. Introduction

Numerous neurodegenerative diseases have been reported to develop neuropathology, including protein aggregate formation within neurons, particularly including aggregates of mutant protein species, such as mutated TDP-43 in amyotrophic lateral sclerosis, amyloid-b in Alzheimer’s disease, a-synuclein in Parkinson’s disease, huntingtin in Huntington’s disease, and ataxin-3 in Spinocerebellar ataxia 3 [1,2,3,4,5]. The prevalence of protein aggregates in neurodegenerative diseases suggests that a protein quality control pathway, such as the macroautophagy (autophagy) pathway, may frequently be impaired or incapable to degrade the accumulation of toxic proteins in these diseases. Autophagy is the main protein quality control pathway that allows the removal of cytoplasmic debris, such as damaged organelles, misfolded protein, or protein aggregates to be recycled [6]. This process occurs through the enclosure of the debris with a double membranous structure known as an autophagosome containing the debris. The complete degradation of the contents of the autophagosome is achieved when lysosomes containing hydrolytic enzymes fuse with autophagosomes to form autolysosomes. Toxic proteins, such as polyQ expanded ataxin-3, which is the cause of spinocerebellar ataxia 3, have been shown to be targeted for degradation through the autophagy pathway [7,8,9,10,11,12,13,14,15].

Spinocerebellar ataxia 3 (SCA3), otherwise known as Machado–Joseph disease, is a neurodegenerative disease affecting the coordination and control of muscles in patients. This autosomal dominant disease is caused by the expansion of the CAG trinucleotide repeat region in the *ATXN3* gene. Whilst the trinucleotide region usually contains between 10–40 trinucleotide repeats, the trinucleotide repeat region within the *ATXN3* gene of SCA3 patients can contain between 40–80 repeats [16,17]. The trinucleotide repeat region encodes for the polyglutamine (polyQ) region of the ataxin-3 protein. Neuropathological staining of SCA3 patient brains at autopsy revealed colocalisation of ataxin-3 with various proteins associated with autophagy. Proteins, such as Atg7, p62, LC3, beclin-1, and lamp2a, are highly expressed in SCA3 patient samples compared to healthy controls and/or colocalised with ataxin-3 within neuronal intranuclear inclusions [18,19,20]. One study has demonstrated that in the presence of an autophagy inhibitor compound, autophagosomes frequently failed to mature in iPSCs derived from SCA3 patients [21].

In this study, we investigated whether the autophagy pathway is impaired within a cell culture model of SCA3 and throughout different ages within a transgenic zebrafish model of SCA3. We also sought to explore the therapeutic potential of compounds that have the capacity to increase activity of the autophagy pathway to provide a beneficial outcome for these SCA3 zebrafish. These compounds included both mTOR-dependent and mTOR-independent inducers of autophagy, including rapamycin, loperamide, trehalose, and MG132. Of these, rapamycin and trehalose have been tested previously on models of SCA3 [8,10,12,22,23]. Previous studies of trehalose treatment of SCA3 HEK293 cells reported that trehalose reduced the number of ataxin-3 positive aggregates [8,12]. Moreover, trehalose treatment of SCA3 mice decreased behavioural deficits, reduced ataxin-3-positive aggregate size, and preserved cerebellar layer thickness [12]. Rapamycin has also been tested previously in various in vitro and in vivo models of SCA3 [10,22,23]. Rapamycin treatment of induced pluripotent stem cells derived from SCA3 patients has been reported to decrease polyQ expanded ataxin-3 levels whilst leaving wild-type ataxin-3 levels unchanged [10]. Within our study, we found that multiple compounds with autophagy modulatory activity were able to rescue the motor impairment of the SCA3 zebrafish. We propose that these findings indicate that compounds with autophagy modulation activity warrant further investigation for the treatment of SCA3.

## 2. Materials and Methods

### 2.1. Generation and Maintenance of SCA3 Cell Culture Model

SH-SY5Y cells were grown in Dilbecco’s Modified Eagle’s Medium (DMEM)/Nutrient Mixture F12 (Thermo Scientific, Waltham, MA USA) and supplemented with 10% fetal bovine serum (FBS, Thermo Scientific). Cells were maintained at 37 °C and 5% CO_2_. A pcDNA3.1 vector containing human ataxin-3 (containing a CAG repeat corresponding to 28Q or 84Q length) or an empty vector control and a neomycin resistant gene were used for stable selection of transfected cells. An amount of 500 µg/mL of neomycin (Sigma Aldrich, Burlington, MA, USA) was used to select for cells stably expressing human ataxin-3. Cells were treated with 250 µg/mL neomycin to maintain expression of the ataxin-3 protein or empty vector control.

### 2.2. Husbandry, Generation, and Maintenance of Transgenic Zebrafish Expressing ATXN3

Experiments carried out using animals in this study were performed in accordance with the animal ethics committee of Macquarie University (ARA: 2016/004 and 2017/019). Zebrafish were housed in a standard recirculating aquarium system at 28.5 °C with a 13-h light and 11-h dark cycle. Transgenic SCA3 zebrafish used in this study had been used and described previously [14]. This transgenic line resulted from crossing driver line zebrafish expressing Tg(*elav3*:Gal4-VP16; mCherry) with responder line zebrafish, Tg(UAS:dsRED,EGFP-ATXN3_Q23) or Tg(UAS:dsRED,EGFP-ATXN3_Q84), to generate zebrafish carrying neuronal expression of human ataxin-3 containing either 23Q or 84Q fused to EGFP. The resulting F1 generation zebrafish were used for the confirmation of autophagy substrates in the adult zebrafish brain (ages 7-month and 18-month). Drug treatment studies utilised the resulting offspring (F2) of in-crossing the F1 generation zebrafish.

### 2.3. Brain and Spinal Cord Extraction from Adult SCA3 Zebrafish

For protein lysate collection, 7- and 18-month-old transgenic SCA3 zebrafish brain and spinal cord were dissected. This involved euthanising zebrafish by immersing them into 4 °C system water for 30 min before commencing dissection. Brain and spinal cord were removed with dissection scissors and fine-tip forceps and directly placed into tissue protein extraction reagent (TPER, Thermo Scientific) containing protease inhibitors (Complete ULTRA Tablets, Roche, Basel, Switzerland). Brain and spinal cord were then homogenised manually using a homogenising dounce and probe sonication.

### 2.4. Drug Treatment of Cell Culture and Zebrafish Models of SCA3

SH-SY5Y cells stably expressing human ataxin-3 (28Q and 84Q) and an empty vector control were seeded into a 24 multi-well plate with a density of 40,000 cells/cm^2^ and incubated at 37 °C and 5% CO_2_., cells were treated with Bafilomycin A1 (Baf A1; 100 nM dissolved in DMSO, Santa Cruz Biotechnology, Dallas, Texas, USA) 70 h after seeding. Cells were treated for 2 h prior to protein extraction. Zebrafish embryos positive for the ATXN3-84Q transgene were identified via the expression of fluorophores (EGFP and dsRED) at 24 h post fertilisation (hpf). Positive embryos were treated with trehalose (50 mM, solubilised in E3 medium), loperamide (16 µM, solubilised in ethanol), rapamycin (1 µM, solubilised in DMSO), MG132 (50 µM, solubilised in ethanol), or chloroquine (3 mM, solubilised in E3 medium). Loperamide, rapamycin, and MG132 were purchased from Cayman Chemicals whilst trehalose and chloroquine were purchased from Sigma Aldrich. These compounds were added to the E3 medium the larvae were incubated in. Control (vehicle) treated animals (all genotypes: non-transgenic siblings, EGFP-ataxin-3 23Q, and 84Q) received the equivalent volume of appropriate vehicle (E3 medium, ethanol, or DMSO). Zebrafish larvae were exposed to a single administration of the compound until 6 days post fertilisation (dpf), where motor behaviour analysis and generation of protein lysates for western blotting were performed. Concentrations were chosen based on the literature used in cell culture studies. Concentrations that produced morphologically abnormal larvae or high mortality were not included in the study. Approximately 20–25 embryos were treated per treatment group per round, and these were merged to represent one replicate.

### 2.5. Motor Behavioural Assay of Zebrafish Larvae

Zebrafish behavioural analysis was performed in the Zebrabox using the Viewpoint Zebralab tracking software. Tracking of 6 dpf larvae was conducted through placement into rows of a 24-well plate, with experimental groups allocated to rows in a randomised manner to eliminate any location bias. The multi-well plate was then acclimatised in the Zebrabox for 20 min. Larvae were then exposed to conditions of 6 min light and 4 min darkness. The total distance travelled in periods of darkness were calculated by the Zebralab software and analysed by the experimenter.

### 2.6. Western Blotting

SH-SY5Y cells stably expressing human ataxin-3 (28Q and 84Q), were washed with ice-cold PBS and incubated in 100 µL RIPA buffer containing protease inhibitors (Complete ULTRA tablets, Roche). Cells were gently agitated on orbital shaker for 5 min and spun down at 18,000 g for 15 min. Zebrafish larvae aged 6 dpf were prepared for protein extraction following euthanasia. Zebrafish larvae were placed into RIPA buffer containing protease and phosphatase inhibitors (Complete ULTRA tablets and PhosphoSTOP tablets, respectively; Roche) and then homogenised manually using a dounce. Equal amounts of protein were separated though SDS-PAGE and transferred onto a 0.45 µm PVDF membrane for immunoblot probing.

Antibodies used included rabbit anti-MJD (kind gift from H. Paulson), rabbit anti-beclin-1 (Proteintech, Rosemont, IL, USA), rabbit anti-p62 (MBL), mouse anti-p62 (Abcam), rabbit anti-LC3B (Abcam), rabbit anti-phosphorylated ULK1 (ser777; Merck Millipore, Burlington, MA, USA), rabbit anti-ULK1 (Cell Signalling Technology, Danvers, MA, USA), and mouse anti-GAPDH (Proteintech). Immunoblots were then probed with the appropriate secondary (Promega, Madison, WI, USA and LiCor Odyssey, Lincoln, NE, USA) and visualized either with chemiluminescence (SuperSignal West Femto Maximum Sensitivity Substrate, Thermo Fisher, Waltham, MA, USA) using the ImageQuant LAS4000 or under fluorescence using the LiCor Odyssey. Band ©ntensity was quantified using Image Studio Lite and the target protein was normalised against the loading control protein (GAPDH).

### 2.7. Statistical Analysis

Data analysis was performed using GraphPad Prism (version 9). Group comparisons, such as ATXN3 genotypes and zebrafish larvae tracking, were analysed using a one-way ANOVA, followed by a Tukey post hoc analysis or mixed-effects analysis. Densitometric analysis of autophagy proteins in the presence of an autophagy inducer versus the vehicle control were compared using a paired Student’s *t*-test. Statistically significant differences are defined as * *p* < 0.05.

## 3. Results

### 3.1. SH-SY5Y Cells Expressing Human Ataxin-3 Demonstrate Autophagy Impairment

**Figure 1:**
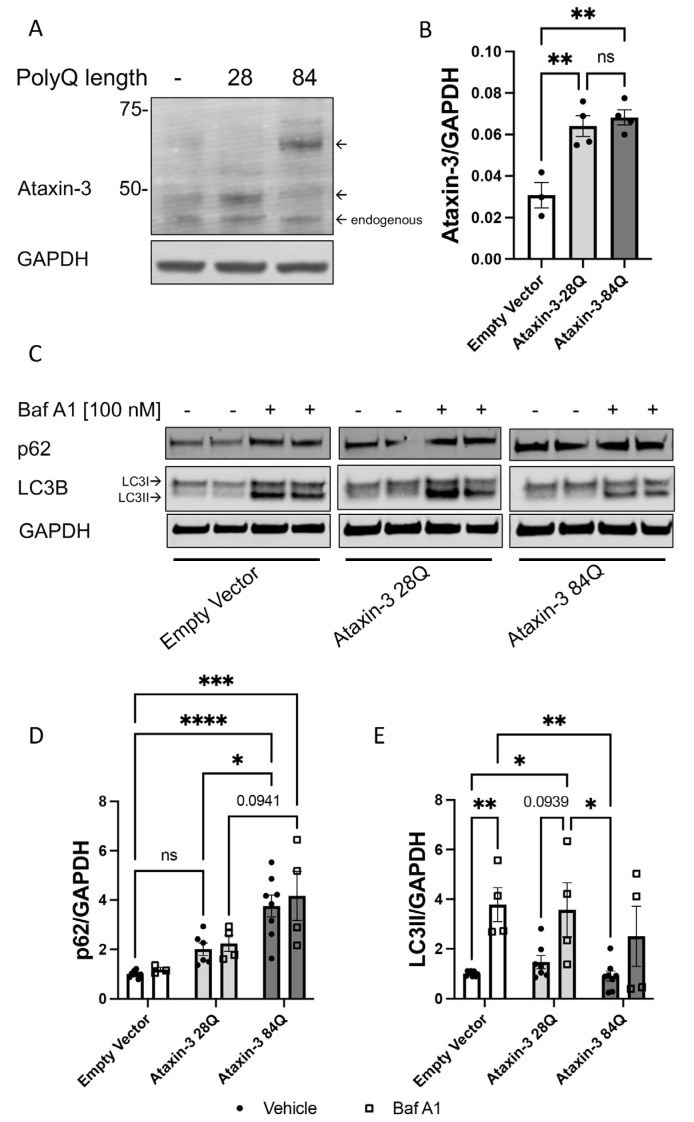
Autophagy impairment identified in a cell culture model of SCA3. (**A**) Western blot of SH-SY5Y cells stably expressing either an empty vector control or human ataxin-3 28Q or human ataxin-3 84Q probed for ataxin-3. (**B**) Quantification of human ataxin-3 levels relative to GAPDH showing increased levels of ataxin-3 in the ataxin-3 28Q and ataxin-3 84Q compared to the empty vector control (*p* = 0.0039 and *p* = 0.0019, respectively, n = 3–4). (**C**) Western blot of SH-SY5Y cells stably expressing either an empty vector control or human ataxin-3 28Q or human ataxin-3 84Q treated with either vehicle (DMSO) or Bafilomycin A1 (Baf A1). Western blot was probed with either p62 or LC3B. (**D**) Baseline levels of p62 were increased in ataxin-3 84Q cells compared to the empty vector control and ataxin-3 28Q cells (*p* < 0.0001 and *p* = 0.0301 respectively, n = 3–8). Baf A1 treatment increased p62 levels in the ataxin-3 84Q cells compared to the empty vector control (*p* = 0.0057).©) LC3II levels did not differ between genotypes at baseline; however, in the presence of Baf A1, empty vector cells had increased LC3II compared to vehicle treated empty vector cells (*p* = 0.0095). Vehicle-treated empty vector cells also had decreased LC3II levels compared to Baf A1-treated ataxin-3 28Q cells (*p* = 0.0193). Additionally, LC3II levels were lower in the ataxin-3 84Q cells treated with vehicle compared to the empty vector and ataxin-3 28Q in the presence of Baf A1 (*p* = 0.0069 and 0.0142 respectively, n = 4–8). Error bars represent mean ± SEM. Statistical analysis performed were a one-way ANOVA and two-way ANOVA followed by Tukey post hoc analysis. * represents *p* < 0.05, ** represents *p* < 0.01, *** represents *p* < 0.001, **** represents *p* < 0.0001.

SH-SY5Y cells stably expressing human ataxin-3 containing 28Q or 84Q were compared with cells expressing empty vector control. We first confirmed that between the genotypes, there was equal expression of human ataxin-3 (Figure 1A,B). We next examined whether expression levels of two widely studied components of the autophagy pathway, p62 and LC3, were altered in protein lysates extracted from the cells expressing the different proteins. At baseline, ataxin-3 84Q cells were observed to have increased p62 and LC3II levels compared to the empty vector or ataxin-3 28Q cells (Figure 1C). Densitometric analysis revealed that, indeed, ataxin-3 84Q cells had increased p62 levels compared to the two genotypes whilst there were no differences in the amount of LC3II between the groups (Figure 1D,E). As autophagy in neuronal cells is a highly dynamic process, levels of autophagy markers are best examined in the presence of an autophagy blocker compound [21,24]. In this study, we inhibited the autophagy pathway using Bafilomycin A1 (Baf A1; 100 nM) in the SCA3 cells. Baf A1 treatment across the genotypes revealed increased p62 levels in the ataxin-3 84Q cells compared to the empty vector control and trending towards significance when compared to the ataxin-3 28Q cells (Figure 1D). When comparing levels of the LC3II, there was an increase between the Baf A1-treated and vehicle-treated empty vector controls, suggesting that autophagy flux was at a high level when the Baf A1 was not present to prevent the autophagosome clearance (Figure 1E). However, in the ataxin-3 expressing cells, regardless of polyQ length, no increase in LC3II levels was produced by the presence of Baf A1, compared to the vehicle treated counterpart. Vehicle treated ataxin-3 84Q cells had decreased LC3II levels compared to Baf A1 treated empty vector and ataxin-3 28Q cells. These results taken together suggest that polyQ expanded ataxin-3 has impaired autophagy dynamics or decreased autophagic flux.

### 3.2. A Transgenic Zebrafish Model of Machado–Joseph Disease Exhibits Autophagy Impairment

Next, we examined whether the autophagy pathway is also perturbed in our transgenic zebrafish model of SCA3. We performed protein extraction from the SCA3 zebrafish of varying ages (6 days fertilisation [dpf], 7 months old, and 18 months old) and performed immunoblotting for markers of autophagy activity. Four different markers that represent different components of the autophagy pathway were examined: ULK1, beclin-1, p62, and LC3B.

**Figure 2:**
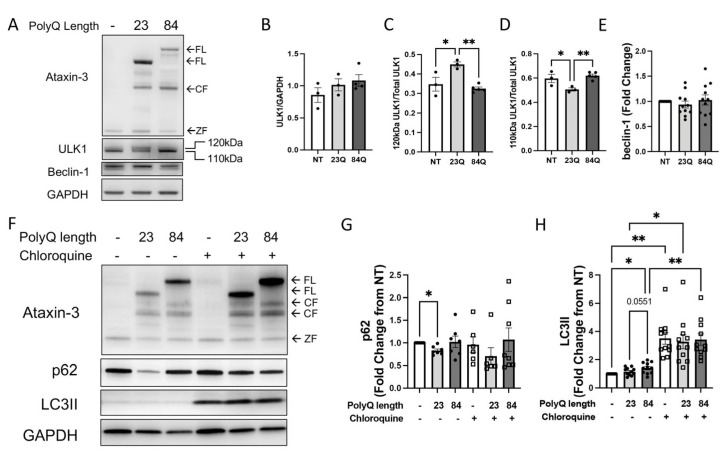
Autophagy impairment identified in SCA3 larvae. (**A**) Representative Western blot of EGFP-ataxin-3 zebrafish larvae aged 6 days post fertilization (dpf) and probed for ULK1 and beclin-1. (**B**) Quantification of ULK1 levels did not have any significant differences. (**C**) Quantification of the 120 kDa ULK compared to total ULK1 showed an increase in the ataxin-3 23Q zebrafish compared to the non-transgenic and ataxin-3 84Q zebrafish (*p* = 0.0181 and *p* = 0.0032, respectively, n = 3–5). (**D**) Quantification of the 110 kDa ULK1 compared to total ULK1 showed a decrease in the ataxin-3 23Q compared to the non-transgenic control and the ataxin-3 84Q zebrafish (*p* = 0.048 and *p* = 0.0083, n = 3–5). (**E**) Quantification of beclin-1 showed no differences between the genotypes (*p* > 0.05, n = 11). (**F**) Representative Western blot of EGFP-ataxin-3 zebrafish larvae treated with either chloroquine (3 mM) or vehicle control between 1–6 dpf. Western blots probed for ataxin-3, p62, and LC3B. (**G**) Quantification of p62 revealed decreased levels in the ataxin-3 23Q genotype compared to the non-transgenic control at baseline (*p* = 0.0171, n = 6–8). (**H**) Quantification of LC3II showed ataxin-3 84Q zebrafish had increased levels compared to the non-transgenic control at baseline (*p* = 0.0454, n = 11). Addition of chloroquine revealed increased LC3II levels compared to the vehicle treatment for each respective genotype (NT: *p* = 0.0013, ataxin-3 23Q: *p* = 0.0148, ataxin-3 84Q: *p* = 0.0044). NT-Non-transgenic, FL-full-length, CF-cleavage fragment, ZF-zebrafish. Error bars represent mean ± SEM. Statistical analysis performed were either paired one-way ANOVA followed by Tukey post hoc analysis or mixed-analysis. * represents *p* < 0.05, ** represents *p* < 0.01.

Immunoblots of the zebrafish larvae at 6 dpf revealed that the zebrafish expressing EGFP-ataxin-3 containing a short polyQ (EGFP-ataxin-3 23Q) had a similar level of human ataxin-3 expression to the zebrafish expressing human ataxin-3 with a long polyQ (EGFP-ataxin-3 84Q), as previously described (Figure 2A) [14]. When we examined levels of ULK1 expression in these lysates, we found that ULK1 existed at two different sizes, 110 kDa and 120 kDa. Interestingly, when quantifying ULK1 bands together, there were no differences between the genotypes (non-significant; Figure 2B). However, when quantifying each band by molecular weight against the total amount of ULK1, it was found that the EGFP-ataxin-3 23Q zebrafish had greater levels of 120 kDa ULK1 and lower levels of 110 kDa ULK1, compared to the other genotypes (Figure 2C,D; *p* < 0.0181 and *p* < 0.0448 respectively, n = 3). To investigate whether the 120 kDa ULK1 band was due to posttranslational modification, such as phosphorylation, we probed our immunoblots for phosphorylated ULK1 (ser777). We identified a 120 kDa phosphor-ULK1 band that varied between the genotypes in a similar manner to the 120 kDa ULK1 band that we had observed (Supplementary Figure S1). Probing for beclin-1 in the SCA3 zebrafish showed no significant difference (Figure 2A,E).

To determine the level of autophagic flux present in the SCA3 zebrafish larvae, we treated the larvae with an autophagy blocker (chloroquine; 3 mM) from 1–6 dpf. These larvae were euthanized for protein lysate extraction and immunoblot analysis for p62 and LC3B levels. Chloroquine inhibits autophagy flux by increasing lysosomal pH, thus preventing the fusion of autophagosomes and lysosomes, making it possible to determine levels of autophagosomes and autophagic cargo due to their degradation being inhibited [25]. From a qualitative perspective, human ataxin-3 levels (cleaved and full-length) were elevated in samples from the transgenic zebrafish following chloroquine treatment when compared to those that were untreated, suggesting that chloroquine is preventing the clearance of ataxin-3 (Figure 2F).

Immunoblotting for p62 appeared to show the lowest amount of p62 in the ataxin-3 23Q group, and this was reflected in the quantification with ataxin-3 23Q zebrafish having lower p62 levels at baseline compared to the non-transgenic control (*p* = 0.0171; Figure 2F,G). Following the addition of chloroquine, we expected to see an increase in p62 levels to signify the inhibition of autophagosome clearance. Interestingly, chloroquine treatment did not result in altered p62 levels (comparing with and without chloroquine for individual genotypes) or between the genotypes (whilst both under chloroquine blockage) (Figure 2G). When immunoblotting for LC3B, we found that at baseline, the Ataxin-3 84Q zebrafish had higher LC3II levels than the non-transgenic zebrafish (*p* = 0.0454). Whilst the amount of LC3II approached a significant difference between ataxin-3 84Q and ataxin-3 23Q zebrafish, a statistically significant difference was not found (*p* = 0.0551; Figure 2H). Chloroquine treatment resulted in increased LC3II levels for each genotype (Figure 2F). Quantification of LC3II in the presence of chloroquine revealed that each genotype had a significant increase when compared to its vehicle control, and there was no difference in LC3II levels between the different genotypes when treated with chloroquine (Figure 2H).

**Figure 3:**
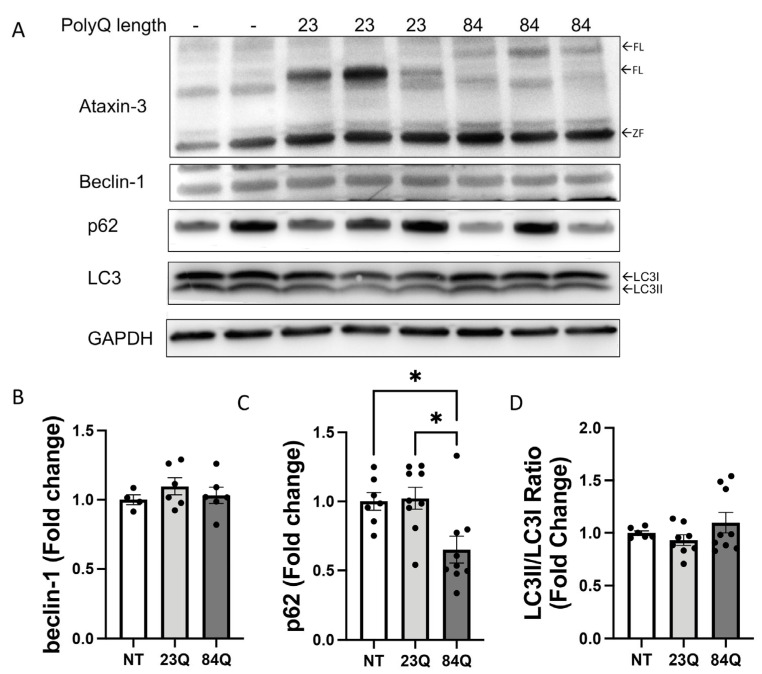
Adult SCA3 zebrafish do not exhibit impairment in the autophagy pathway. (**A**) Representative Western blot of 7-month-old SCA3 zebrafish brain and spinal cord protein samples. Western blot was probed with beclin-1, p62, and LC3B. (**B**) Quantification of beclin-1 revealed no significant differences between the genotypes (n = 4–6). (**C**) Quantification of p62 revealed a decrease in the ataxin-3 84Q zebrafish compared to the ataxin-3 23Q zebrafish and the non-transgenic control (*p* = 0.0106 and *p* = 0.0254, respectively, n = 7–9). (**D**) Quantification of LC3II/LC3I ratio showed no significant differences between the genotypes (n = 6–9). NT-Non-transgenic, FL-full-length, ZF-zebrafish. Error bars represent mean ± SEM. Data points represent individual adult zebrafish Statistical analysis performed was a one-way ANOVA followed by a Tukey post hoc analysis. * represents *p* < 0.05.

As SCA3 is an aging disease, autophagy may be impaired later on throughout the lifespan of the zebrafish. Therefore, we examined whether protein levels of autophagy substrates were altered in the transgenic SCA3 zebrafish at later ages. Brain and spinal cord protein samples were prepared from the different transgenic SCA3 zebrafish genotypes for Western blotting. We found that the zebrafish continued to express human ataxin-3 at 7-months-old (Figure 3A). However, ULK1 protein was not detected upon Western blotting in the SCA3 zebrafish brain samples. When comparing the expression levels of the various markers of autophagy, beclin-1, p62, and LC3B, there was no strong indication of changes in the mutant ataxin-3 fish compared to the wild-type ataxin-3 and non-transgenic control zebrafish samples (Figure 3A). This was confirmed upon quantification of the beclin-1, LC3II/LC3I levels (Figure 3B,D). Interestingly, levels of p62 were decreased in the adult ataxin-3 84Q zebrafish compared to those carrying wild-type human ataxin-3 and non-transgenic controls (Figure 3C).

**Figure 4:**
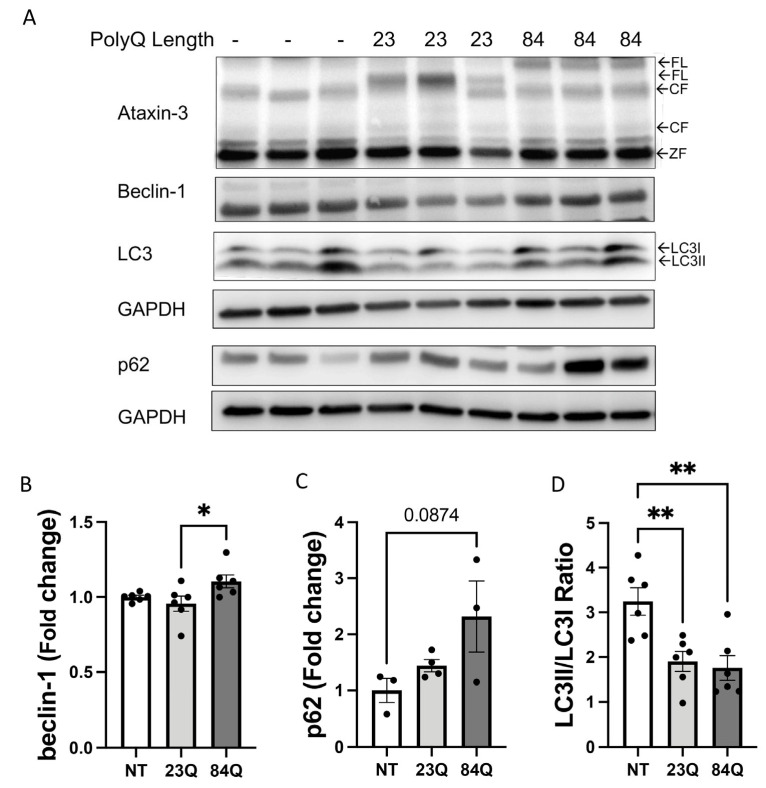
Aging SCA3 zebrafish exhibit autophagy impairment. (**A**) Representative Western blot of EGFP-ataxin-3 zebrafish brain and spinal cord protein samples at 18 months of age. Western blot was probed with autophagy markers beclin-1, p62, and LC3B. (**B**) Quantification of beclin-1 revealed increased levels in the ataxin-3 84Q group compared to ataxin-3 23Q zebrafish (*p* = 0.0415, n = 6). (**C**) Quantification of p62 showed a trend of increased p62 levels in the ataxin-3 84Q zebrafish compared to the non-transgenic control (*p* = 0.0874, n = 3–4). (**D**) Quantification of the LC3II/LC3I ratio revealed that expression of the human ataxin-3, regardless of polyQ length, decreased levels compared to the non-transgenic control (ataxin-3 23Q: *p* = 0.0068, ataxin-3 84Q: *p* = 0.0046, n = 6). NT-Non-transgenic, FL-full-length, CF-cleavage fragment; ZF-zebrafish. Error bars represent mean ± SEM. Data points represent individual adult zebrafish. Statistical analysis performed was a one-way ANOVA followed by Tukey post hoc analysis. * represents *p* < 0.05, ** represents *p* < 0.01.

Expression of autophagy markers in the brain and spinal cords of the various genotypes of zebrafish at 18-months-old (corresponding with advanced age) were next assessed. Transgenic SCA3 zebrafish at this age were expressing human ataxin-3 (Figure 4A). Similar to the 7-month-old samples, ULK1 protein was not detected in the SCA3 zebrafish brain samples. Immunoblotting for beclin-1 appeared to differ when comparing against the genotypes (Figure 4A). Quantification of beclin-1 revealed that the ataxin-3 84Q fish had higher levels of beclin-1 compared to the ataxin-3 23Q zebrafish brain only (Figure 4B). Levels of p62 showed an elevation in the ataxin-3 84Q fish compared to the wild-type ataxin-3 and non-transgenic controls. However, a densitometric analysis of p62 levels confirmed a trend in the polyQ-dependent increase, but this did not reach statistical significance (Figure 4C; *p* = 0.0874). The LC3II/LC3I ratio decreased with the expression of human ataxin-3 23Q or 84Q compared to the non-transgenic controls (Figure 4A,D). These results suggest that aging SCA3 zebrafish, or even zebrafish expressing human ataxin-3, wild type, or polyQ expanded, show impairment in autophagy dynamics at the stage of autophagosome maturation (decreased LC3II/LC3I ratio) and a decreased ability to degrade the contents within the autophagosomes.

### 3.3. Drug Compound Screening for Autophagy Inducers on SCA3 Zebrafish Ameliorates Motor Impairement

**Figure 5:**
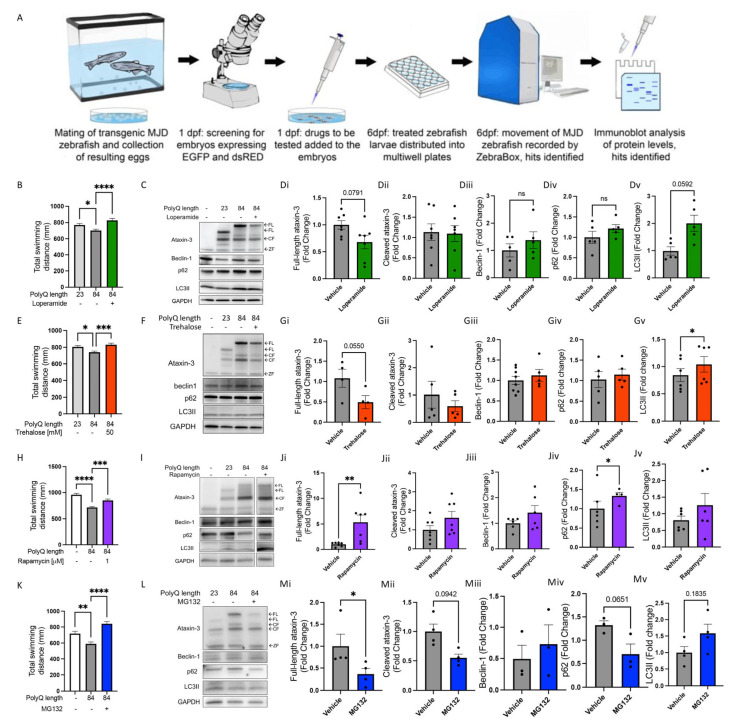
Known autophagy inducers rescue motor behaviour in transgenic SCA3 zebrafish without signs of autophagy induction. (**A**) Schematic of workflow of performing drug treatment studies in the SCA3 zebrafish. (**B**) Quantification of motor behaviour assay with and without loperamide (16 µM) treatment. (**C**) Representative Western blot of SCA3 zebrafish with and without loperamide treatment and probed for ataxin-3, beclin-1, p62 and LC3B. (**Di**–**Dv**) Quantification of human ataxin-3 (full-length and cleaved fragments), beclin-1, p62, and LC3II in SCA3 zebrafish with and without loperamide treatment. (**E**) Quantification of motor function assay of the SCA3 zebrafish when exposed to trehalose (50 mM). (**F**) Representative Western blot of SCA3 zebrafish treated with vehicle versus trehalose and probed for ataxin-3, beclin-1, p62, and LC3B. (**Gi**–**Gv**) Quantification of human ataxin-3 (full-length and cleaved fragments), beclin-1, p62, and LC3II. (**H**) Quantification of motor behaviour assay of the SCA3 zebrafish when exposed to rapamycin (1 µM). (**I**) Representative Western blot of SCA3 zebrafish with and without rapamycin treatment and probed for ataxin-3, beclin-1, p62, and LC3B. (**Ji**–**Jv**) Quantification of human ataxin-3 (full-length and cleaved fragments), beclin-1, p62, and LC3II. (**K**) Quantification of motor behaviour assay in the SCA3 zebrafish when exposed to MG132 (50 µM). (**L**) Representative Western blot of SCA3 zebrafish treated with MG132 and probed with ataxin-3, beclin-1, p62, and LC3B. (**Mi**–**Mv**) Quantification of human ataxin-3 (full-length and cleaved fragments), beclin-1, p62, and LC3II. NT-Non-transgenic, FL-full-length, CF-cleavage fragment ZF-zebrafish. Error bars represent mean ± SEM. Data points represent one experimental replicate consisting of 20–25 embryos per experiment. Statistical analysis performed was either a one-way ANOVA followed by Tukey post hoc analysis or paired Student’s *t*-test. * represents *p* < 0.05, ** represents *p* < 0.01, *** *p* < 0.001 and **** represents *p* < 0.0001.

In our investigation, we found that SCA3 models, including our transgenic zebrafish model, exhibit altered autophagy function. Therefore, we sought to examine whether treating the SCA3 zebrafish larvae with known autophagy inducers described in the literature (loperamide, trehalose, rapamycin, and MG132) would have beneficial effects [8,10,23,26]. Our drug treatment pipeline consisted of treating the SCA3 zebrafish at 24 hpf with either loperamide (16 µM), trehalose (50 mM), rapamycin (1 µM), or MG132 (50 µM) until 6 dpf where motor behaviour and levels of human ataxin-3 (full-length and cleaved) and markers of autophagy (beclin-1, p62, and LC3II) were assessed (Figure 5A).

Loperamide, an FDA approved compound that acts via blocking Ca^2+^ channels, had a beneficial effect on the swimming behaviour of 6 dpf zebrafish, with vehicle treated ataxin-3 84Q fish swimming shorter distances compared to the ataxin-3 23Q fish and loperamide treatment increasing the distance swum by the ataxin-3 84Q zebrafish (Figure 5B; *p* = 0.047 and *p* < 0.0001, respectively, n = 112–125). Western blotting of human ataxin-3 levels suggested that loperamide treatment had decreased the amount of ataxin-3 present compared to the vehicle treated control (Figure 5C). However, densitometric analysis revealed no significant differences in the amount of full-length (*p* = 0.0791) or cleaved ataxin-3 in the samples from loperamide treated larvae (Figure 5(Di,Dii)). Similarly, loperamide treatment did not affect the amount of autophagy markers such as beclin-1, p62, or LC3II (Figure 5(Diii–Dv)).

The next candidate that we tested, trehalose, is a disaccharide thought to activate the autophagy pathway via its activation of transcription factor EB (TFEB) within lysosomes [27]. Treatment with 50 mM trehalose increased the distance swum by the ataxin-3 84Q zebrafish at 6 dpf compared to vehicle treated ataxin-3 84Q (Figure 5E; *p* = 0.0005, n = 104–208), resulting in similar distances travelled by the treated ataxin-3 84Q larvae to that of the ataxin-3 23Q. Immunoblotting for human ataxin-3 showed a modest decrease in levels of full-length and cleaved ataxin-3 after trehalose treatment (Figure 5F). Densitometric analysis revealed that the decrease in full-length ataxin-3 with trehalose treatment did not reach significance (*p* = 0.0550), and no differences between vehicle and treated samples in the amount of cleaved ataxin-3 were present (Figure 5(Gi,Gii)). Whilst there were similarities in the amount of beclin-1 and p62 before and after treatment with trehalose (Figure 5(Giii,Giv)), LC3II levels increased with trehalose treatment (Figure 5(Gv); *p* = 0.012).

Rapamycin, an mTOR inhibitor compound well known to induce mTOR-dependent autophagy, was tested at 1 µM between 1–6 dpf in the SCA3 zebrafish [24]. Rapamycin treatment produced an increased distance travelled by the SCA3 zebrafish compared to that seen in the vehicle treated ataxin-3 84Q group (Figure 5H, *p* = 0.0101, n = 84–118). Interestingly, rapamycin treatment increased full-length ataxin-3 levels without affecting levels of cleaved ataxin-3 levels (Figure 5I,(Ji,Jii); *p* = 0.0187 and *p* = 0.1305, respectively, n = 7). When comparing the levels of autophagy markers, rapamycin treatment appeared to affect all autophagy substrates (Figure 5I). However, densitometric analysis revealed beclin-1 and LC3II levels were similar between vehicle and rapamycin treatment and p62 levels increased with rapamycin treatment (Figure 5(Jiii,Jv); *p* = 0.0353, n = 4).

Lastly, MG132 is well known to be an inhibitor of the ubiquitin proteasome system (UPS) [28]. It has been proven that MG132 also activates the autophagy pathway as a compensatory mechanism resulting from the inhibition of the UPS [29,30,31]. Treatment with MG132 had a positive effect on the motor function of the SCA3 zebrafish at 6 dpf, producing increased distances swum compared to the vehicle treated SCA3 larvae (Figure 5K; *p* < 0.0001, n = 91–96). MG132 treatment was able to reduce full-length human ataxin-3 84Q levels (*p* = 0.0477) whilst cleaved ataxin-3 levels was not affected (Figure 5L,(Mi,Mii); *p* = 0.0942, n = 4). Western blotting of the autophagy substrates, beclin-1 or LC3II, did not appear to be affected with MG132 treatment, and this was confirmed with quantification. However, there was a trend towards decreased p62 levels with MG132 treatment in the SCA3 zebrafish compared to the vehicle control (Figure 5(Miii–Mv); *p* = 0.0651, n = 3).

## 4. Discussion

### 4.1. Autophagy Impairment in Spinocerebellar Ataxia 3

In this study, we examined baseline levels of different autophagy-related proteins within a SH-SY5Y model of SCA3 and transgenic SCA3 zebrafish at a range of ages. We firstly found signs of impairment to autolysosome formation or autophagic flux in the SH-SY5Y cells stably expressing human ataxin-3 84Q. We found that levels of the autophagy substrate p62 were higher in cells expressing ataxin-3 84Q than in those expressing empty vector or ataxin-3 28Q, suggesting that p62 was not being efficiently cleared in the cells expressing ataxin-3 84Q. We also found that cells expressing human ataxin-3 (both wild-type and mutant) exhibited a smaller increase in LC3II in response to treatment with an autophagy inhibitor, suggesting that the cells expressing human ataxin-3 had a lower level of autophagic flux. These results are consistent with previous reports, in which aberrant levels of autophagic flux are identified in cells from SCA3 patients in the presence of an autophagy blocker [21]; however, a difference between cells expressing wild-type human ataxin-3 and an empty vector was a surprise.

We next examined baseline levels of autophagy-related proteins in SCA3 zebrafish at larval zebrafish (6 dpf), adult, and aged stages. Within the larval stage, we found that zebrafish larvae expressing ataxin-3 23Q exhibited a higher abundance of a 120 kDa ULK1 protein, and lower abundance of a 110 kDa ULK1 protein compared to that found in larvae expressing ataxin-3 84Q or non-transgenic controls. ULK1 is a protein important in the formation of autophagosomes [6,32]. Vasconcelos-Ferreira et al. (2022) have recently reported decreased ULK1 transcript levels in samples from SCA3 patients and murine models of SCA3 [33]. We hypothesise that the two ULK1 isoforms identified within our study are the ULK1 protein (110 kDa) and ULK1 with a post-translational modification (PTM), such as phosphorylation (120 kDa). This finding is qualitatively supported by probing the immunoblots for phosphorylated ULK1. In line with this finding, we also observe that larvae expressing human ataxin-3 23Q have lower levels of p62 compared to both the non-transgenic and ataxin-3 84Q zebrafish. Together these findings suggest that expression of wild-type human ataxin-3 (with non-expanded polyQ length) may play a role in increasing upstream autophagy activity and that decreased phosphorylated ULK1 could be another marker of decreased autophagy activity in the SCA3 zebrafish larvae [32]. Interestingly, no differences in levels of p62 or LC3II were found between the genotypes, following treatment with chloroquine, suggesting that the downstream effects of this ULK1 phenotype are either masked or overcome by the autophagy blocker. Unfortunately, we were not able to detect any ULK1 protein in the brain and spinal cord of the adult and aging SCA3 zebrafish. This finding is in line with Huang et al., 2016 report of low mRNA *ulk1a* levels in the zebrafish brain at 4 months of age, compared to other organs, such as the heart and intestines [34].

Within the ataxin-3 84Q zebrafish larvae we also observed higher LC3II levels at baseline than that in non-transgenic zebrafish. This finding suggests that the larvae expressing ataxin-3 84Q may be exhibiting some impairment in the formation of autolysosomes, resulting in accumulation of LC3II, rather than dynamic clearance of LC3II within autophagic flux. However, it should be noted that in the zebrafish larva studies, LC3II levels were compared against a loading control (GAPDH), rather than as a ratio over its unconjugated form, LC3I. This is because within our larval samples, LC3II levels were readily detectable via western blotting, whereas LC3I levels were often not. This is in line with previous reports that LC3II levels can sometimes be more readily detected than LC3I levels, due to instability of LC3I protein in freeze thaw cycles, a stronger affinity of LC3II binding to PVDF membranes, and better protein separation with higher percentage polyacrylamide gels in SDS-PAGE [24,35].

We then investigated the level of autophagy markers in brain samples from adult and aged transgenic SCA3 zebrafish. These life stages were defined as 7-months versus 18-months of age due to the relatively shorter lifespan of the zebrafish of approximately 2–3 years [36]. We found that signs of autophagy impairment were lacking in the brains of 7-month-old adult SCA3 zebrafish. The only significant difference seen within the different autophagy markers at that age was decreased levels of p62 in the ataxin-3 84Q zebrafish in comparison to ataxin-3 23Q zebrafish and non-transgenic controls. A decreased presence of p62 can be a sign of either increased autophagy activity, resulting in p62 clearance, or decreased expression of p62 in the animals. This is in contrast to the aged zebrafish revealing a trend towards increase in p62 levels in the ataxin-3 84Q group compared to the non-transgenic animals. We hypothesise that this difference is because defective autophagy may not be apparent until the later age. In the aged 18-month-old zebrafish, we found that the ataxin-3 84Q zebrafish exhibited higher levels of beclin-1 than that present in the zebrafish expressing ataxin-3 23Q. This finding is in contrast with previous reports of SCA3 patient brain samples exhibiting decreased levels of beclin-1 [19,21]. Elevated levels of beclin-1 are often found when autophagy has been induced. However, it has been reported previously that elevated beclin-1 levels can occur in response to the presence of toxic protein species and therefore a requirement for autophagy induction in models of neurodegenerative diseases [37,38]. Earlier reports describe caspase-dependent cleavage of beclin-1 within cells and these fragments had the propensity to shift the activation of the autophagy pathway to the apoptotic pathway [39]. In the 7-month- and 18-month-old zebrafish, a smaller band (37 kDa) below the full-length beclin-1 (51 kDa) was noted, but protein levels did not vary between the genotypes (data not shown). Beclin-1 proteolysis within these SCA3 zebrafish may be another avenue to explore in determining baseline autophagy levels.

In the 18-month-old zebrafish, we also found that both ataxin-3 23Q and ataxin-3 84Q zebrafish exhibited lower LC3II/I ratios than non-transgenic control zebrafish. This finding is suggestive that both transgenic zebrafish lines had decreased levels of autophagic flux than that of the non-transgenic controls. This finding is also in line with our finding that that the SHSY5Y cells expressing human ataxin-3 (either ataxin-28Q or ataxin-3-84Q) had lower autophagic flux than those expressing just an empty vector. These findings are consistent with previous reports of autophagy impairment in samples from end-stage SCA3 patients [18,19,20]; however, the effect of wild-type ataxin-3 on autophagy function requires further investigation.

Nascimento-Ferreira et al. reported signs of impaired autophagy function in SCA3 patient brains at autopsy back in 2011. They found that in patient brain samples, particularly within the putamen, showed an increased number of puncta stained with autophagy substrates p62, LC3, and Atg16, compared to healthy control patients. They also found that beclin-1 levels were reduced in SCA3 patient fibroblasts [19]. Since then, several studies have shown evidence of autophagy impairment within SCA3 patient brain samples [18,20,21,33]. Future studies to examine immunohistochemical co-staining of the brains of aging SCA3 zebrafish for autophagy machinery and ataxin-3 would be valuable.

One limitation of our study of baseline autophagy markers is the lack of an autophagy inhibitor compound to confirm the presence of autophagy impairment in the adult zebrafish. Our in vitro and in vivo (larval) SCA3 models utilised Bafilomycin A1 and chloroquine, two known autophagy blockers, to obtain that insight. Bafilomycin A1 and chloroquine are known to inhibit the fusion between autophagosomes and autolysosomes, therefore preventing the clearance of cytoplasmic debris [24,25]. Within the SCA3 zebrafish model, zebrafish larvae were treated with chloroquine due to their ability to be semi-permeable and thus easily absorb compounds when placed in their aquatic environment. However, adult zebrafish do not have this advantage and instead require ingestion of the drug compound. Further complications arise when considering the feasibility of treatment. This includes the amount of treatment used per fish per day on a circulating water system. However, to be definitive in these conclusions of the presence of autophagy impairment in the aged SCA3 zebrafish, it would be best practise to treat the zebrafish with an autophagy blocker compound, such as chloroquine, before euthanasia to determine what the levels of the autophagy markers are in a non-dynamic setting [24].

### 4.2. Autophagy-Inducing Compounds as a Treatment for Spinocerebellar Ataxia 3

Following our findings of differences in autophagy function in the SCA3 zebrafish at larval stages, we next sought to investigate the therapeutic benefit of known autophagy inducing compounds within the SCA3 zebrafish. Numerous studies have previously demonstrated the beneficial effects of overexpressing autophagy substrates or treating models of SCA3 with autophagy inducing compounds, and so, we sought to test for beneficial effects of these treatments regardless of baseline autophagic function [8,10,11,12,14,19,23,33,40,41]. We tested known mTOR-dependent and -independent autophagy compounds used previously in models of SCA3 and related neurodegenerative diseases [8,10,23,26].

We found that treatment with loperamide, trehalose, rapamycin, and MG132 each produced significant improvements in the distance swum by the SCA3 zebrafish. When probing for markers of autophagy following these treatments, we found that treatment with trehalose had produced a significant increase in LC3II, which may be indicative of successful autophagy induction by the treatment. Nevertheless, to convincingly demonstrate that the treatment had induced autophagy, cotreatment with the compound and an inhibitor of autolysosome formation, is required. In a similar manner to this, we found that treatment with rapamycin had produced an increased level of p62 within the treated zebrafish larvae. This finding is surprising because the induction of autophagy would usually be thought to decrease the amount of p62 present due to autophagic clearance. However, it is possible that the treatment with rapamycin had instead increased levels of p62 through transcriptional regulation to aid autophagic processing [42].

Our findings of the beneficial effects of the trehalose treatment on SCA3 are in line with previous reports of beneficial effects of trehalose treatment on cell culture and mouse models of SCA3 [8,12]. Trehalose has also been trialled clinically for safety and efficacy in SCA3 patients. Whilst minor adverse events were reported, findings were suggestive of a stabilisation of disease progression [43]. Similarly, the beneficial effect of rapamycin treatment on the swimming of the SCA3 zebrafish was in line with previous reports of beneficial effect of rapamycin on various models of SCA3 [10,23]. Treatment of SCA3 mice with the rapamycin analogue, temsirolumis or CCI-779, has also been reported to produce improvements in motor behaviour, a reduction in cytoplasmic ataxin-3 protein, and ataxin-3 positive aggregates [23]. Nevertheless, co-treatment of a mouse model of SCA3 with temsirolimus and lithium chloride together did not produce beneficial effects on motor function; despite signs of autophagy induction in the treated SCA3 mouse brain, there were decreased levels of ataxin-3 protein and insoluble ataxin-3 protein aggregates [22].

Treatment with loperamide has been previously reported to aid removal of protein aggregates in zebrafish models of Huntington’s disease [26], but it has not been previously examined in SCA3 models. Williams et al. (2008) found that treatment with loperamide reduced levels of both soluble and insoluble polyQ expanded huntingtin protein [26]. Moreover, screening of compound libraries showed loperamide to be an autophagy inducing compound that increased LC3II levels and reduced polyQ expanded protein [44]. Whilst loperamide treatment is useful for proof of principal demonstration of the potential cellular benefits of inducing autophagy, it has lower translational potential due to poor ability to cross the blood–brain barrier and psychoactive effects when delivered centrally [45].

The beneficial effect of treatment with MG132, seen through improvements in the swimming of the SCA3 zebrafish and removal of the human ataxin-3 84Q protein, was surprising. MG132 is primarily used as an inhibitor of the UPS [28]. However, UPS inhibition has also been shown to induce the autophagy pathway as a compensatory mechanism [29,30,31]. We speculate that the concentration and treatment duration of MG132 tested in our study was sufficient to induce autophagy. This finding suggests that, at least in the SCA3 zebrafish, the ubiquitin proteasome system, which is inhibited by MG132, is not a major pathway of ataxin-3 degradation. It also indicates that such an indirect method of autophagy induction may warrant further investigation for the treatment of the disease.

It is worth noting that whilst these compounds have been previously reported to induce autophagy activity, within this study, it was not confirmed whether the administered doses were indeed inducing autophagy through co-treatment with an autophagy blocker. Whilst interpretations were generated by analysing baseline levels of p62 and LC3II following these treatments, examining the effect of co-treatment with a known autophagy inhibitor, such as chloroquine, is warranted [24].

In conclusion, we report that autophagy impairment occurs in both a SH-SY5Y model of SCA3 and in our zebrafish model of the disease. We report that treating our SCA3 zebrafish with various compounds with autophagy induction capacity was able to produce improved swimming of the zebrafish. These findings suggest that autophagy function requires further investigation in models of the disease.

## Data Availability

Not applicable.

## Supplementary Materials

The following supporting information can be downloaded at https://www.mdpi.com/article/10.3390/cells12060893/s1, Figure S1: Identification of a post-translational modification of ULK1 in the transgenic SCA3 zebrafish.
