# Supplementary material for: Autophagy Function and Benefits of Autophagy Induction in Models of Spinocerebellar Ataxia Type 3
Source: Cells. 2023 Mar 14;12(6):893. doi: 10.3390/cells12060893 (PMC10047838; doi:10.3390/cells12060893)
Supplement: Supplementary file 1 [file cells-12-00893-s001.zip › cells-2131818-supplementary.pdf]

Supplementary Figure S1 – Ulk1 vs phosphorylated ULK1

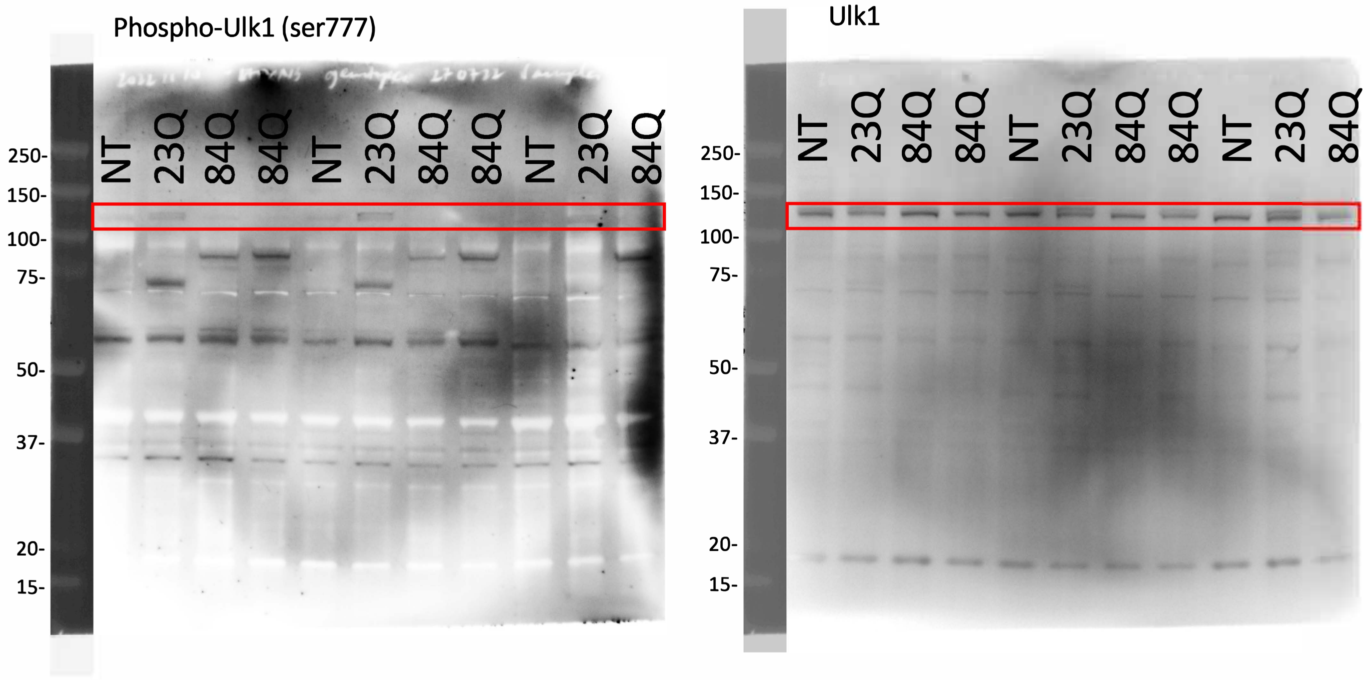

Supplementary Figure S1. Identification of a post-translational modification of ULK1 in the transgenic SCA3 zebrafish. Protein samples of SCA3 zebrafish larvae aged 6 days post fertilization were immunoblotted and probed for phospho-ULK1 (ser777) and ULK1. Highlighted in red, the bands indicating phospho-ULK1 overlap with the 120 kDa band seen in the ULK1 membrane.
